# STATE OF THE EVIDENCE FOR MULTIVITAMIN/MULTIMINERAL SUPPLEMENTATION AND COGNITIVE AGING

**DOI:** 10.1093/geroni/igae098.3052

**Published:** 2024-12-31

**Authors:** Rachael Patusco, Ankita Srivastava

**Affiliations:** Haleon, Warren, New Jersey, United States; WNS Global Services, Gurugram, Haryana, India

## Abstract

In 2017, nearly 40% of Americans aged 60 years and over consumed a multivitamin/multimineral (MVM) product. Nutrient inadequacies, for which older adults are at greater risk, can negatively influence cognitive aging. Bringing together the converging themes of MVM popularity and cognitive aging, this review synthesized evidence on the effects a broad spectrum MVM has on cognitive aging. A literature search was conducted using ProQuest (Embase, Medline) and PubMed. Seven studies met inclusion criteria. All studies were double-blind, placebo-controlled, randomized clinical trials utilizing an MVM product (range = 10 - 26 essential nutrients). Duration of interventions ranged from 6 months to 12 years across 63 to 5,974 participants aged 60 years and older from the U.S. and European countries. There was heterogeneity in cognitive assessments utilized with some overlap of tested domains, such as episodic memory & executive function. Shorter and smaller trials (≤ 1 year, n=3), found no overall effect of MVM supplementation on cognitive performance, however some domains were sensitive to MVM intervention. Among longer duration trials (≥ 3 years, n=4), 75% reported significant improvements in episodic memory & global cognition. When measured across studies, MVM supplementation significantly improved serum vitamin levels, which is consistent with broader MVM supplementation research. Despite heterogeneity in treatment effects in shorter duration trials, in longer duration trials (>1 year), MVMs can elucidate beneficial effects, suggesting dose & duration may contribute to the treatment effect. Evidence gaps exist in exposing mechanism of action.

